# Measurement of blood pressure in rats: Invasive or noninvasive methods?

**DOI:** 10.14814/phy2.70041

**Published:** 2024-09-12

**Authors:** Viktória Kapsdorferová, Soňa Grešová, Pavol Švorc

**Affiliations:** ^1^ Department of Medical Physiology, Faculty of Medicine Pavol Jozef Safarik University Kosice Slovakia

**Keywords:** blood pressure, heart rate, rat, reference values

## Abstract

Experiments should always be based on control values. This assumption fully applies to cardiovascular parameters, such as heart rate (HR) and blood pressure (BP), which are highly sensitive to various external and internal stimuli and can already be significantly altered when an experiment begins. Therefore, it is necessary to determine which values are defined as a starting point (i.e., control and baseline) or compare them with valid reference values if the goal is to evaluate the changes after experimental intervention. A generally accepted principle is a reciprocal relationship between BP and HR, in which one parameter affects the other and vice versa. BP can be measured using two methods—noninvasively (tail‐cuff) and invasively (telemetry, direct measurements of BP after introducing the sensor directly into the artery), and HR directly or by extrapolation from BP recordings. This study does not aim to evaluate the results of individual studies, but to review whether there are differences in control (baseline) BP values in normotensive and hypertensive male rats using invasive versus noninvasive methods, and to investigate whether there is a causal relationship between BP and HR in in vivo experiments with male rats.

## 1 INTRODUCTION

Among critics and reviewers, methods of blood pressure (BP) measurement, whether invasive or noninvasive (tail‐cuff method), which are most suitable for accurate determination of BP in small laboratory animals, such as rats or mice, remains controversial. Various methods have their advantages and disadvantages (Table 1). Based on recommendations for BP measurement in experimental animals by the American Heart Association in 2005, Harrison et al. (2024) reported that the choice of method(s) should be primarily based on the objectives of the study.

**TABLE 1:** Advantages and disadvantages of radiotelemetry and tail‐cuff method for blood pressure measurement, as outlined by Harrison et al. (2024).

Method	Advantage(s)	Disadvantages
Radiotelemetry	Continuous and direct measurement of blood pressure and evaluation of changes over time	Expensive refurbishment (after two or three uses) of telemetry transmitters and represents permanent costs Implantation of telemetry transmitters is technically demanding and requires surgical skill Special conditions for the placement of animals Animals should be completely anesthetized Implantation of a telemetry transmitter is invasive, and recovery is required (at least 10 days) until stabilization of hemodynamic parameters
Tail‐cuff	Noninvasive, less expensive, and does not require special surgical skills	Before actual measurement, animals must be trained for 2–3 days Animals are restrained and heated, which can induce a stress response(s) Does not enable the evaluation of hemodynamic changes during the day or the free movement of animals, and the measurement of diastolic blood pressure is not optimal Measurement values reflect the average pressure over several minutes and do not reflect transient pressure fluctuations

Over the past 20 years, investigators have attempted to noninvasively measure BP in mice and rats, with varying degrees of success. The ability to accurately and noninvasively measure systolic and diastolic BP (SBP and DBP, respectively), except for the heart rate (HR) and other blood flow parameters in rodents, is of great clinical value to researchers. Noninvasive measurement methods have gained popularity in recent years. However, many factors in choosing noninvasive technique(s) must be considered, including heating and restriction (Yen et al., 1978). Harrison et al. (2024) proposed several general recommendations regarding when and how to use the tail‐cuff technique.

Invasive BP measurements use telemetry or direct catheterization of an artery (Van Vliet et al., 2000). Invasive methods are considered the most physiologically representative in rats, and some consider them the gold standard because they provide the most accurate values. However, its use requires surgical intervention (Plehm et al., 2007). Although invasive measurements provide baseline BP, there are occasional fluctuations due to anesthesia that interfere with normal BP (Parasuraman & Raveendran, 2012).

In the 1980s, clinical chronobiologic studies demonstrated that BP appeared to be an independent variable of the cardiovascular system. In cases of suspected or confirmed failure of autonomic regulation of the cardiovascular system, as well as at fixed HR after pacemaker implantation in patients with complete atrioventricular block, the circadian rhythm of BP persists (Mann et al., 1984). Accordingly, it has been hypothesized that there are autonomic control mechanisms for HR and BP (Davies et al., 1984). These conclusions were supported in a study by Portaluppi et al. (1991), who found that, even in subjects with decompensated heart defects, the circadian rhythm of BP persisted.

Similarly, experimental studies involving rats point to the possible independence of BP from HR. For example, after lesioning the suprachiasmatic nuclei in the hypothalamus, the group 24‐h rhythm of SBP was eliminated, while a significant group circadian rhythm was detected for HR (Stoynev et al., 1996). Sei et al. (1997) reported that diurnal changes in mean arterial pressure (MAP) in rats are a result of the waking rhythm and that the mechanism of diurnal control of MAP may differ from that of HR or body temperature. Significant changes in arterial BP were observed in rats exposed to electromagnetic pulses, while HR was not altered (Li et al., 2007). Averages over the entire series of consecutive measurements revealed significantly increased SBP and DBP in restrained rats after mild isoflurane anesthesia; however, HR was not affected (Hem et al., 2019). In spontaneously hypertensive rats (SHR), BP and physical activity increased with age, but HR decreased. In normotensive rats, neither BP nor physical activity exhibited significant changes, but HR decreased with age (Kohno et al., 1994). The greater rise in BP upon arousal in SHR is not associated with differences in HR or changes in activity and may be part of the underlying mechanisms that contribute to hypertension in SHR (Head et al., 2004).

These results suggest that BP and HR are probably mutually independent variables of the cardiovascular system, but they can significantly influence one another. Although the control or baseline values of BP and HR are described in the methods sections of these studies, as well as changes in values after the experimental intervention, their causal relationship is rarely, if ever, described. The question is, if an experimental intervention changes BP, will HR change accordingly, or will it behave as an independent variable and respond to the intervention independently of BP?

From a physiological perspective, in mammals, BP affects HR and, conversely, HR affects BP. This is one of the regulatory interrelationships between HR and BP. In other words, if BP is increased, HR decreases and, thus, participates in compensating for elevated BP and vice versa. It logically follows that there should be a negative causal relationship between HR and BP. However, if HR increases, BP increases and vice versa—therefore, a positive relationship. Thus, it remains an open question whether there is a causal relationship between BP and HR in rats already at the start of the experiment according to different BP measurement methods.

The aim of this study was not to critically evaluate the results of individual studies but to review whether there are differences in control (baseline) BP values in normotensive and hypertensive sexually mature male rats using invasive versus noninvasive methods reported in 2023 and 2024, and to investigate whether there is a causal relationship between BP and HR in in vivo experiments with sexually mature male rats reported from 2021 to 2024.

## 2 METHODS

This study followed the Preferred Reporting Items for Systematic Review and Meta‐Analyses (PRISMA) statement (see Data S1 for PRISMA 2020 Checklist).

### 2.1 Eligibility criteria

The PICOS framework (Population, Intervention, Comparator, Outcomes, Study Design) guided the identification of inclusion criteria, with the following specifications: (1) Population: studies with sexually mature (determined either by weight 250–350 g or age 3–4 months) normotensive or hypertensive (clearly described BP status; all SBP values exceeding 140 mmHg were classified under the hypertensive group) male rats of any strain, with clearly described numerical BP control values (not graphical) in awake animals before the experiment, or baseline numerical BP values from awake animals that went through the preparatory phase of the experiment (the experimental intervention itself was not applied), and with numerical HR values that were determined together with at least one BP parameter (SBP, DBP, or MAP); (2) Intervention: studies that reported BP measurements using non‐invasive (tail‐cuff) or invasive (telemetry, direct measurements via catheterization); (3) Comparator: differences between normotensive and hypertensive sexually mature male rats when using invasive or noninvasive methods of BP measurement; (4) Outcomes: differences in control (baseline) BP values and causal relationship between BP and HR; (5) Study design: experimental studies.

### 2.2 Search strategy

Due to the considerable heterogeneity of published data regarding BP measurement in male rats, the primary focus was on assessing differences between noninvasive versus invasive in vivo methods. A manual search of references was conducted in the Web of Science database (P.Š.). This study examined all available numerical BP values reported from 2023 to 2024, using the keyword “blood pressure in rats,” to assess whether there are differences in measured BP when using the tail‐cuff method versus invasive methods. To answer our second question, whether there is a causal relationship between BP and HR in rats already at the start of the experiment itself according to different methods of BP measurement, we used control values for BP (SBP, DBP, and MAP) and HR from studies registered in the Web of Science database from 2021 to 2023, using the same keyword “blood pressure in rats.” The search was conducted in April 2024 and was not limited by language.

### 2.3 Study selection

The selected studies were independently manually screened based on the inclusion criteria, as defined in the eligibility criteria, by two authors (P.Š. and S.G.).

To assess whether there are differences in measured BP when using the tail‐cuff method versus invasive methods, the extracted BP data (SBP, DBP, and MAP) came from studies that met the following criteria:

- Only sexually mature male rats were included (determined either by weight 250–350 g or age 3–4 months)
- Clearly described BP status; more specifically, normotensive, or hypertensive males
- All SBP values exceeding 140 mmHg were classified under the group of hypertensive rats
- Clearly described numerical control values for BP in awake animals before the experiment using the tail‐cuff method in normotensive male rats (Ahad et al., 2022; Ahmed et al., 2023; Alanazi et al., 2023; Baka et al., 2023; Candido et al., 2023; Chen et al., 2023; Desplanche et al., 2023; Forester et al., 2024; Fu et al., 2023; Gomes, de Moura, et al., 2023; Gonçalves et al., 2023; Gonsalez et al., 2023; Goto et al., 2023; Khazaeli et al., 2023; Kocaman Kalkan et al., 2023; Kolesnyk et al., 2023; Lim et al., 2024; Ma et al., 2024; Maneesai et al., 2023; Matsumoto et al., 2023; McCalla et al., 2024; Mohammed et al., 2023; Moke et al., 2023; Monteiro et al., 2023; Shamardl et al., 2023; Shen et al., 2023; Teng et al., 2023; Wang et al., 2023; Yuan et al., 2023; Zhang et al., 2024) and hypertensive male rats (Candido et al., 2023; Castoldi et al., 2023; Chen et al., 2023; Corrêa et al., 2023; D'Ambrosio et al., 2023; Gonçalves et al., 2023; Jo et al., 2023; Lim et al., 2024; Liskova et al., 2023; Liu et al., 2023; Mohammed et al., 2023; Tandirerung & Krisna, 2023; Wang et al., 2023; Yan et al., 2023; Yuan et al., 2023).
- Or baseline BP values from awake, normotensive male rats that went through the preparatory phase of the experiment, but the experimental intervention itself was not applied using invasive methods (carotid artery (Olatoye et al., 2023; Raji et al., 2023; Wu et al., 2023); telemetry (Gomes, Batista, et al., 2023; Toczek et al., 2023; Zhang et al., 2023); abdominal aorta (Mohamed et al., 2024; Mohammed Abdulsalam et al., 2023)) or hypertensive male rats (telemetry (Fan et al., 2024; Geraldes et al., 2023; Toczek et al., 2023) and abdominal aorta (Sun et al., 2024)).

To answer our second question, whether there is a causal relationship between BP and HR in rats already at the start of the experiment itself according to different methods of BP measurement, we used control values for BP (SBP, DBP, and MAP) and HR from studies, in which sex, sexual maturity (determined either by weight 250–350 g or age in months 3–4), BP status (normotensive or hypertensive), and BP and HR measurements (invasive or non‐invasive) were explicitly described. Only HR values that were determined together with at least one BP value were considered. BP and HR values of newborn offspring, females, and older animals were excluded from the study. The development of BP and HR was also not analyzed. The animals were classified into four groups, according to the initial (control) BP and the method of measurement:

- Group 1 consisted of male rats with normotensive BP values (tail‐cuff method) (Aali et al., 2021; Afzal et al., 2021; Ahmad, 2022; Ahmed et al., 2023; Alam et al., 2021; Amer et al., 2022; Anamalley et al., 2022; Badr et al., 2021; Baka et al., 2023; Baskaran et al., 2022; Batool et al., 2022; Bian et al., 2021; Bin Jardan et al., 2021; Candido et al., 2023; Chia et al., 2021; Coatl‐Cuaya et al., 2022; Dantas et al., 2021; Del Mauro et al., 2021; Desplanche et al., 2023; Draginic et al., 2022; Fauss et al., 2021; García‐Pedraza et al., 2022; Gonsalez et al., 2023; Grigorova et al., 2022; Hashemi et al., 2021; Hashmi et al., 2021; Hong et al., 2022; Hsieh et al., 2021; Huang et al., 2022; Iampanichakul et al., 2022; Ito et al., 2021; Jan‐On et al., 2021; Lei et al., 2023; Lezama‐Martinez et al., 2021; Li et al., 2022; Liu et al., 2021; Luo et al., 2021; Maneesai et al., 2022, 2023; Mendes et al., 2022; Moke et al., 2023; Nguelefack‐Mbuyo et al., 2022; Nguyen et al., 2022; Ojetola, Adedeji, & Fasanmade, 2021; Ojetola, Adeyemi, David, et al., 2021; Pan et al., 2021; Pauziene et al., 2023; Prasad et al., 2022; Shamardl et al., 2023; Simko et al., 2022; Sunagawa et al., 2021; Torok et al., 2021; Wang et al., 2023; Zou et al., 2022)
- Group 2 was hypertensive according to the tail‐cuff method (Afzal et al., 2021; Ajamu et al., 2021; Baskaran et al., 2022; Bian et al., 2021; Bin Jardan et al., 2021; Candido et al., 2023; Coatl‐Cuaya et al., 2022; El Maleky et al., 2021; Hsieh et al., 2021; Kluknavsky et al., 2022; Lee et al., 2021; Lezama‐Martinez et al., 2021; Liskova et al., 2023; Liu et al., 2021; Luo et al., 2021; Matsuoka et al., 2021; Nakatsukasa et al., 2022; Pan et al., 2021; Pauziene et al., 2023; Rassler et al., 2022; Simko et al., 2022; Soltani Hekmat et al., 2021; Wang et al., 2023)
- Group 3 was normotensive, however, BP was measured using an invasive method, such as telemetry, telemetry in the abdominal aorta, femoral artery, and distal aorta; direct measurements of BP were made using catheterization of the abdominal aorta, femoral artery and carotid artery (Griffiths et al., 2021; Selejan et al., 2022; Zhang et al., 2023), telemetry in the abdominal aorta (Ayaz et al., 2021; Barrera et al., 2021; Potter et al., 2021), in femoral artery (Gomes, de Moura, et al., 2023) and in the distal aorta (Das et al., 2022); direct measurements of BP were made using catheterization in the abdominal aorta (Costa‐Ferreira et al., 2021; Oliveira et al., 2021; Silva et al., 2021), in femoral (Barretto‐de‐Souza et al., 2021; Cruz et al., 2021; dos Santos et al., 2021, 2022; Fioretti et al., 2022; Flahault et al., 2021; Kirillov et al., 2021; Lopes et al., 2022; Luz et al., 2022; Oliveira et al., 2022; Sedighi et al., 2021; Sharma et al., 2021), and in the carotid artery (Komnenov & Rossi, 2023; Olatoye et al., 2023; Zicha et al., 2021)
- Group 4 was hypertensive males, in which BP was also measured using invasive methods, such as telemetry (Griffiths et al., 2021; Selejan et al., 2022), or catheterization of the femoral (Bandoni et al., 2021; Gardim et al., 2021; Marc et al., 2021) or and carotid arteries (Oleksa et al., 2021)

In the latter two groups, the animals underwent a recovery phase, the length of which was determined by BP stabilization.

### 2.4 Data extraction

P.Š. performed initial manual data extraction using Microsoft Word® software. The following data were collected from selected studies: mean HR and BP data (SBP, DBP, and MAP), author, year of publication, rat strain, number of evaluated rats according to the inclusion criteria, as well as the method of BP measurement (see Data S2 and S3 for details). In the second data extraction phase, V.K. and S.G. independently checked the correctness of individual collected data and verified their accuracy.

### 2.5 Assessment of risk of bias

The aim of this study was not to critically evaluate the results and methodological quality of individual studies. As a result, we did not assess the risk of bias using Cochrane's Risk of Bias Tool or any other validated scoring system. Instead, we only focused on a statistical analysis of extracted mean HR and BP data.

### 2.6 Statistical analysis

BP data reported as baseline or control values were summed, and averages, along with other statistical parameters, were calculated. An unpaired nonparametric Mann–Whitney test was used to analyze averaged BP data from 2023 to 2024; a *p* < 0.05 was required for significance. In normotensive and hypertensive male rats, SBP, DBP, and MAP were compared between noninvasive and invasive BP measurement methods. The differences in SBP, DBP, and MAP between normotensive Sprague–Dawley (SD) and Wistar/Wistar‐Kyoto (WKY) rat strains using the tail‐cuff method were also compared. Statistical comparison between other strains and BP measurement methods was not conducted due to fewer studies (see Data S2 for details). Statistical analysis and figure generation were performed by V.K. using Prism 8 software (GraphPad, San Diego, CA, USA). It is important to note that most studies described changes in BP only in graphical form or merely noted changes in BP without providing numerical values.

For the analysis of the causal relationship between BP and HR, we used Pearson's correlation test to calculate the correlation coefficients. Correlation coefficients were calculated from the immediate control, or baseline, values of BP and HR by P.Š. using Microsoft Excel® software. Because not all BPs were assessed together with HR in every study, the number of correlated values is reported in parentheses. Based on the interpretation of the Cohen correlation coefficient, we considered *r* = −0.3 to −0.5, large; *r* = −0.5 to −0.7, very large; *r* = −0.7 to −0.9 and *r* = −0.9 to −1.0, almost perfect; moderately positive dependence, *r* = 0.3 to 0.5, large; *r* = 0.5 to 0.7, large; *r* = 0.7 to 0.9, very large; and *r* = 0.9 to 1.0, almost perfect.

## 3 RESULTS

### 3.1 Study selection

**FIGURE 1:**
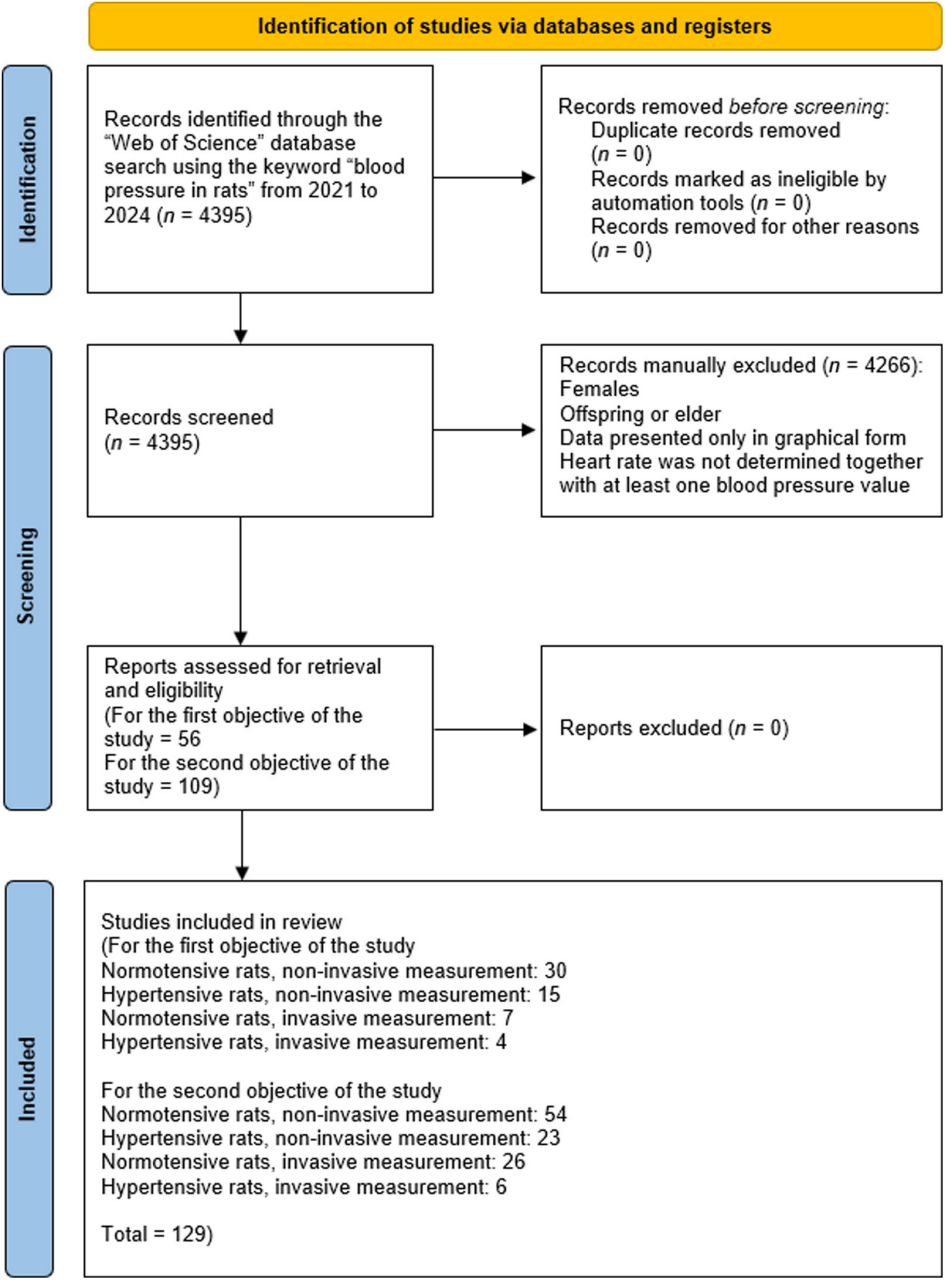
PRISMA 2020 flow diagram for systematic reviews which included searches of databases and registers only. This study aimed to review whether there are differences in control (baseline) blood pressure values in normotensive and hypertensive sexually mature male rats using invasive versus noninvasive methods reported in 2023 and 2024 (first objective of the study), and to investigate whether there is a causal relationship between blood pressure and heart rate in in vivo experiments with sexually mature male rats reported from 2021 to 2023 (second objective of the study). Adapted from Page et al. (2021).

The initial number of records identified through the Web of Science database from 2021 to 2024 using the keyword “blood pressure in rats” was 4395. This study examined all available numerical BP values reported from 2023 to 2024 to assess whether there are differences in measured BP when using the tail‐cuff method versus invasive methods (first objective of the study). To assess a causal relationship between BP and HR in rats (second objective of the study), we used control values for BP (SBP, DBP, and MAP) and HR from 2021 to 2023. No duplicates, records marked as ineligible by automation tools, or records removed for other reasons were removed (*n* = 0). After manual screening of selected studies, a total of 4266 studies were excluded based on the exclusion criteria (females, offspring, or elder individuals, data presented only in graphical form, HR was not determined together with at least one BP value). The remaining 129 studies were assessed for retrieval and eligibility for the first (*n* = 56 out of 129) and second (*n* = 109 out of 129) objective of the study, with some studies contributing to both objectives of the research. For the first objective, the studies comprised with normotensive rats (*n* = 30) and hypertensive rats (*n* = 15) undergoing noninvasive BP measurement, along with normotensive rats (*n* = 7) and hypertensive rats (*n* = 4) undergoing invasive BP measurement. For the second objective, the studies involved normotensive rats (*n* = 54) and hypertensive rats (*n* = 23) with noninvasive BP measurement and normotensive rats (*n* = 26) and hypertensive rats (*n* = 6) with invasive BP measurement (see Figure 1 for details).

### 3.2 Comparison of control (baseline) BP values in normotensive and hypertensive rats using invasive or noninvasive BP measurement methods

**FIGURE 2:**
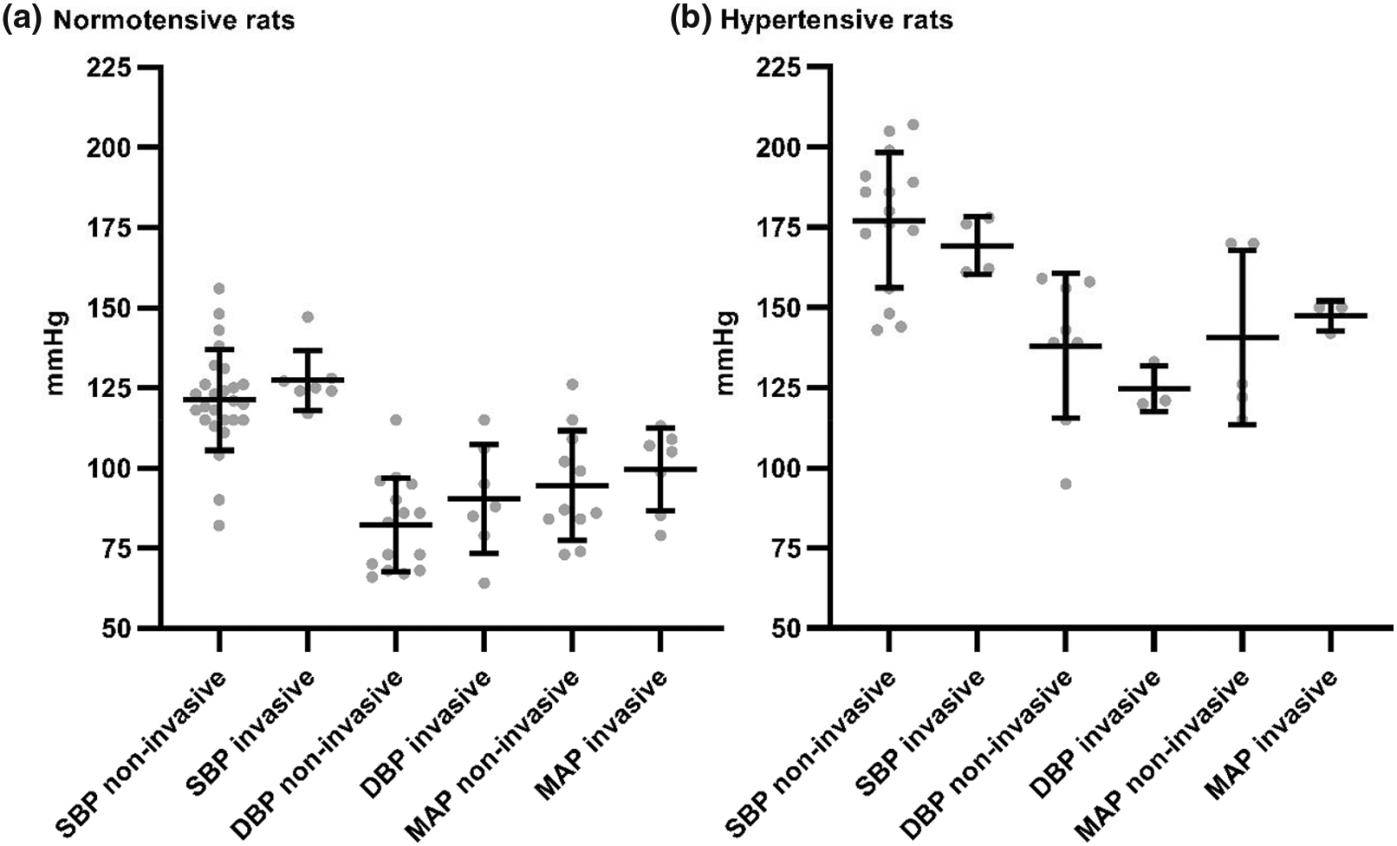
Graphical presentation of mean blood pressure values measured using noninvasive (tail‐cuff) and invasive methods in (a) normotensive, and (b) hypertensive male rats reported in 2023 and 2024.The data are displayed as individual data points with mean line ± standard deviation for each group. DBP, diastolic blood pressure; MAP, mean arterial pressure; SBP, systolic blood pressure.

The Hodges–Lehmann calculation of differences in medians and the lack of significance in the Mann–Whitney U test indicated that the selection between noninvasive and invasive methods may not significantly affect the overall outcomes in both normotensive and hypertensive male rats (see Table 2 and Figure 2 for details). The limited statistical power can be influenced by the differences in sample sizes between BP measurement methods. The *p*‐values ranged from 0.1583 to 0.7143 (SBP in normotensive rats [difference between medians (Hodges–Lehmann): 4.5, lower and upper 95% CI for noninvasive method: 114.9 and 127.5, lower and upper 95% CI for invasive methods: 118.8 and 136.1, Mann–Whitney U: 58.5, *p* = 0.1583]); DBP in normotensive rats (difference between medians [Hodges‐Lehmann]: 10.00, lower and upper 95% CI for noninvasive method: 74.16 and 90.24, lower and upper 95% CI for invasive methods: 74.57 and 106.0, Mann–Whitney U: 40, *p* = 0.3983); MAP in normotensive rats [difference between medians (Hodges–Lehmann): 6.00, lower and upper 95% CI for noninvasive method: 82.95 and 106.0, lower and upper 95% CI for invasive methods: 87.68 and 111.4, Mann–Whitney U: 31.5, *p* = 0.5505]; SBP in hypertensive rats (difference between medians [Hodges–Lehmann]: −11.5, lower and upper 95% CI for noninvasive method: 165.4 and 188.8, lower and upper 95% CI for invasive methods: 154.9 and 183.6, Mann–Whitney U: 21.5, *p* = 0.4241); DBP in hypertensive rats (difference between medians [Hodges–Lehmann]: −19.00, lower and upper 95% CI for noninvasive method: 119.1 and 156.9, lower and upper 95% CI for invasive methods: 106.7 and 142.6, Mann–Whitney U: 6, *p* = 0.2606); MAP in hypertensive rats (difference between medians [Hodges–Lehmann]: 20.00, lower and upper 95% CI for noninvasive method: 106.9 and 174.3, lower and upper 95% CI for invasive methods: 135.9 and 158.8, Mann–Whitney U: 6, *p* = 0.7143).

**TABLE 2:** Blood pressure data from normotensive and hypertensive male rats were obtained using noninvasive and invasive methods reported in 2023 and 2024.

	SBP (mmHg)	DBP (mmHg)	MAP (mmHg)
Normotensive rats			
Non‐invasive	121.2 ± 15.66	82.2 ± 14.53	94.45 ± 17.12
	*n* = 26	*n* = 15	*n* = 11
Invasive	127.4 ± 9.325	90.29 ± 16.99	99.54 ± 12.82
	*n* = 7	*n* = 7	*n* = 7
*p*‐value	0.1583	0.3973	0.5505
Hypertensive rats			
Non‐invasive	177.1 ± 21.10	138 ± 22.58	140.6 ± 27.13
	*n* = 15	*n* = 8	*n* = 5
Invasive	169.3 ± 8.995	124.7 ± 7.234	147.3 ± 4.619
	*n* = 4	*n* = 3	*n* = 3
*p*‐value	0.4241	0.2606	0.7143

*Note*: The data are presented as mean ± standard deviation for each group and were analyzed using unpaired nonparametric Mann–Whitney test; *p* < 0.05 was required for significance. Non–invasive—awake or freely moving animals, in which blood pressure was measured using a noninvasive method (tail‐cuff method) before the experiment itself. Invasive—awake or freely moving animals, in which the animals were preprepared (catheterization, implantation of telemetry sensors, and the like) for the ongoing experiment, but without subsequent targeted experimental intervention, disregarding the place and method of measurement (telemetry, carotid artery, femoral artery, and abdominal aorta). In the group of animals in which blood pressure was measured invasively, the animals went through a recovery phase, the length of which was determined by blood pressure stabilization.

Abbreviations: DBP, diastolic blood pressure; MAP, mean arterial pressure; *N*, number of measurements from which the average value was calculated; SBP, systolic blood pressure.

Based on the analysis, there are no significant differences in SBP, DBP, or MAP between the normotensive SD and WKY rat strains when using the tail‐cuff method. However, it is essential to consider the limitations, such as the number of studies conducted with each strain, which may influence the interpretation of the results SBP between SD and WKY strain (difference between medians (Hodges–Lehmann): −1.73, lower and upper 95% CI for SD strain: 114.3 and 129.0, lower and upper 95% CI for WKY strain: 109.2 and 124.2, Mann–Whitney U: 48.5, *p* = 0.5021); DBP between SD and WKY strain (difference between medians [Hodges–Lehmann]: 4.510, lower and upper 95% CI for SD: 60.90 and 88.70, lower and upper 95% CI for WKY: 69.90 and 89.28, Mann–Whitney U: 12, *p* = 0.8636); MAP between SD and WKY strain (difference between medians [Hodges–Lehmann]: −8.36, lower and upper 95% CI for SD: 73.69 and 117.8, lower and upper 95% CI for WKY:75.17 and 100.3, Mann–Whitney U: 8, *p* = 0.3152).

### 3.3 Interrelationship between blood pressure and heart rate under in vivo experimental conditions

When using a noninvasive method of measuring BP in normotensive and hypertensive male rats, control values did not exhibit any dependence on HR (Table 3). Hypothetically, this could mean that the contribution of HR to changes in BP need not be considered. Because experiments are mostly performed during working hours―more specifically, in the light (inactive) period of the rats' regimen day―it would only apply to this period. Of interest may be whether there is a relationship between BP and HR during the dark (i.e., active) period.

**TABLE 3:** Correlation coefficients between control SBP, DBP, MAP, and HR in normotensive and hypertensive male rats and using noninvasive and invasive methods of BP measurement reported in selected studies from 2021 to 2023.

Rats	HR – SBP	HR – DBP	HR – MAP
Normotensive – noninvasive	*r* = −0.1 (*n* = 47)	*r* = −0.26 (*n* = 27)	*r* = −0.06 (*n* = 23)
Hypertensive – noninvasive	*r* = 0.13 (*n* = 18)	*r* = 0.27 (*n* = 14)	*r* = 0.03 (*n* = 11)
Normotensive – invasive	***r* = −0.57** (*n* = 9)	***r* = 0.52** (*n* = 8)	***r* = 0.31** (*n* = 24)
Hypertensive – invasive	***r* = 0.87** (*n* = 4)	***r* = 0.97** (*n* = 4)	***r* = 0.61** (*n* = 8)

*Note*: *n* = number of pairs from which the correlation coefficient was calculated. Significant values determined by Pearson's correlation test are highlighted in bold. Based on the interpretation of the Cohen correlation coefficient, we considered *r* = −0.3 to −0.5, large; *r* = −0.5 to −0.7, very large; *r* = −0.7 to −0.9 and *r* = −0.9 to −1.0, almost perfect; moderately positive dependence, *r* = 0.3 to 0.5, large; *r* = 0.5 to 0.7, large; *r* = 0.7 to 0.9, very large; and *r* = 0.9 to 1.0, almost perfect.

Abbreviations: DBP, diastolic blood pressure; HR, heart rate; MAP, mean arterial pressure; SBP, systolic blood pressure.

Using an invasive method of measuring BP in normotensive rats, a negative dependence between BP and HR, and a positive correlation between DBP, MAP, and HR, were found. Thus, increasing HR decreases SBP but increases DBP and MAP. A statistically significant positive relationship between all BPs and HR was found in hypertensive rats (Table 3). HR, at invasive measurement of BP in normotensive rats, was higher compared with tail‐cuff values (365.05 ± 35.53 beats/min vs 355.33 ± 42.53 beats/min) in contrast to hypertensive rats (346.5 ± 34.37 beats/min vs 382.02 ± 50.99 beats/min). If BP changes after an experimental intervention and HR significantly participates in these changes, then subsequent changes in, for example, the electrophysiological parameters of the heart, which are dependent on HR, must also be taken into account, which can significantly affect the final result.

## 4 DISCUSSION

Comparisons of SBP, DBP, and MAP between noninvasive and invasive methods did not demonstrate significant differences in both normotensive and hypertensive rats, as well as between normotensive SD and WKY rat strains when using the tail‐cuff method. The lack of statistical significance could be indicative of limited statistical power due to the relatively small sample sizes and discrepancy in sample sizes, respectively in the number of studies, between BP measurement methods. A possible explanation for the variation in the number of studies may be the reliability of BP measurements. Overall, SBP is considered the most reliable parameter in noninvasive tail‐cuff measurements due to the underlying measurement principle. Conversely, telemetry, which is capable of detecting decreases in pulse pressure, typically identifies MAP as the most reliable parameter. Prior research often reports only SBP for noninvasive measurements and solely MAP for invasive measurements (see Data S2 and S3 for details). This situation indicates a fundamental limitation in comparing these two methods of BP measurement, as they prioritize different BP values as the most reliable.

Given the lack of significant results, we can only hypothesize that when using invasive methods of BP measurement in normotensive male rats, it is necessary to account for potentially higher control or baseline values present at the beginning of the experiment. The opposite tendency was observed in hypertensive male rats, in which SBP and DBP were not significantly lower when using invasive methods of BP measurement. The extent to which these values correspond to actual BP in sexually mature male rats is controversial. Interventions and animal handling are stressful factors that can affect BP. For instance, the noninvasive tail‐cuff method for measuring BP is a reliable, simple, and cost‐effective technique. However, it necessitates restraining the rat, warming it, and applying pressure to its tail, which constitutes a stress factor that may potentially influence the outcomes of BP measurements (Harrison et al., 2024; Kubota et al., 2006; Lipták et al., 2017). The question arises whether hypertensive rats respond more significantly to stress induced by the tail‐cuff method compared to normotensive rats, potentially explaining why noninvasive BP measurements in hypertensive rats show higher values in both SBP and DBP than invasive measurements (see Table 2), or whether another mechanism is involved. Elevated BP in hypertensive animal models may result from a natural physiological response to stress induced by confinement in a plastic cage during measurement, or even due to the gender of the handler—rodents exhibit a strong physiological stress reaction to male odor. Nonetheless, these observations remain inconclusive, as rats may respond to acute stress without a corresponding increase in BP parameters (Lipták et al., 2017).

One of the other limitations of this study is the exclusion of female rats. There is a paucity of experimental studies involving female rats, particularly in relation to our specific research questions and the selected time period of the available literature on which this study is focused. As illustrated in Tables 2 and 3, there is a significant discrepancy even among the sample sizes of the male groups. We anticipate that the inclusion of female rats would exacerbate these disparities, further compromising the statistical power and validity of the results. Therefore, it is essential to emphasize the increased need for including both normotensive and hypertensive female rats in future experimental research, particularly in assessing BP differences between invasive and noninvasive methods.

Moreover, it is necessary to consider the time required for recovery when using invasive methods. The BP values obtained may be similar to the actual pressure, but only in the inactive phase of the rats' regimen day because experiments are, in large part, performed during the day. In general, the specification of the light/dark cycle is often inadequately detailed or entirely missing in many studies. This lack of precise timing for experimental interventions presents a limitation, as it neglects the potential impact of circadian variability on the parameters being studied. Among the studies employing the tail‐cuff method that were included in our systematic review, only 8% offered a detailed account of the 12:12 light/dark cycle and the precise timing of the experimental intervention (Dantas et al., 2021; Huang et al., 2022; Kluknavsky et al., 2022; Ma et al., 2024; Moke et al., 2023; Teng et al., 2023; Zhang et al., 2024). Notably, 27% of the studies did not provide any information concerning the light/dark cycle or the timing of the experimental intervention (Ahad et al., 2022; Ahmad, 2022; Afzal et al., 2021; Bin Jardan et al., 2021; Desplanche et al., 2023; El Maleky et al., 2021; Forester et al., 2024; García‐Pedraza et al., 2022; Goto et al., 2023; Hashmi et al., 2021; Hong et al., 2022; Ito et al., 2021; Kolesnyk et al., 2023; Lei et al., 2023; Lezama‐Martinez et al., 2021; Liu et al., 2023; Matsumoto et al., 2023; Nakatsukasa et al., 2022; Ojetola, Adedeji, & Fasanmade, 2021; Rassler et al., 2022; Soltani Hekmat et al., 2021; Sunagawa et al., 2021; Tandirerung & Krisna, 2023; Zou et al., 2022). This issue weakens the interpretation of the BP data, particularly when comparing values obtained using the tail‐cuff method at unspecified times (even if they were collected during the same light/dark period) within the rats' daily cycle, with values obtained from 24‐h telemetry measurements. Additionally, many studies average several values without considering the exact time of day when the individual values were taken, which raises questions about the relevance of such averages from a chronobiologic perspective. It is crucial for future experimental studies to provide detailed information about the implemented light/dark cycle, including the exact duration and timing of light/dark phases, as well as the specific timing of experimental interventions.

Potential variability in the measured BP values may also result from factors such as breeding species, weight, age, method of animal handling, experimental conditions, and other factors. Therefore, our results should be interpreted with caution. Further research with larger sample sizes, considering the abovementioned factors, is necessary to verify the differences between noninvasive and invasive methods for BP measurement in both normotensive and hypertensive rats.

Based on the results, we can only conclude that HR and BP probably behave as two independent parameters in the cardiovascular system, which would exclude the assessment of mutual relations between HR and BP, but only when using a noninvasive method of measuring BP. Using the invasive method of BP measurement, the relationships between BP and HR are statistically significant, but were probably influenced by the small number of data points evaluated. It is essential to acknowledge that HR responses to external stress are generally more pronounced than the associated changes in BP. Thus, similar to BP, it is crucial to interpret our findings concerning HR with great caution, considering the potential impact of stress factors. Consequently, more accumulation of data is needed to objectively assess the relationship(s) between BP and HR, taking into account the aforementioned limitations of experimental studies.

## 5 CONCLUSION

Conclusions drawn from this study can only be hypothetical, and informative, and do not indicate the actual reference values of BP in rats. Our study did not find significant differences in BP measurements between noninvasive and invasive methods in both normotensive and hypertensive male rats, suggesting that the selection between noninvasive and invasive methods may not significantly affect the overall BP outcomes. However, we hypothesize that invasive methods of BP measurement in normotensive male rats may require accounting for potentially higher baseline values at the experiment's onset, whereas in hypertensive male rats, SBP and DBP are not significantly lower when using invasive methods. Additionally, our findings suggest that HR and BP may behave independently in the cardiovascular system when using noninvasive methods. The relationship between BP and HR remains uncertain with invasive methods due to the small number of data points evaluated. Currently, well‐defined “normal” BP ranges in rats are still not available. The existence of circadian BP rhythms should also be considered when into reference values. Relatively few studies have addressed BP changes over a 24‐h period, and it has been established that BP displays a circadian rhythm. As such, it is necessary to design standardized experiments, primarily using radiotelemetry, to monitor dynamic changes in BP depending on the time of day, sex, and age of the animals.

## FUNDING INFORMATION

None.

## CONFLICT OF INTEREST STATEMENT

We declare that none of the authors has any conflict of interest.

## ETHICS STATEMENT

This systematic review does not contain any experimental work with animals performed by any of the authors.

## REVIEW REGISTRATION STATEMENT

We declare that this systematic review was not registered in any prospective registry before its initiation. As a result, there is no preexisting protocol available for this systematic review.

## Supporting information


Data S1.

Data S2.

Data S3.
