# Supplementary material for: Measurement of blood pressure in rats: Invasive or noninvasive methods?
Source: Physiol Rep. 2024 Sep 12;12(17):e70041. doi: 10.14814/phy2.70041 (PMC11392657; doi:10.14814/phy2.70041)
Supplement: Supplementary file 2 — Data S2. [file PHY2-12-e70041-s002.docx]

**Supplement 2** – Baseline values of BP data (SBP, DBP and MAP) from normotensive and hypertensive sexually mature male rats were obtained using non-invasive or invasive methods reported in 2023 and 2024. BP, blood pressure; SBP, systolic blood pressure; DBP, diastolic blood pressure; MAP, mean arterial pressure; n, number of evaluated rats for mean BP data; SD, Sprague-Dawley rat; SHR, Spontaneously Hypertensive rat; WKY, Wistar or Wistar-Kyoto strain;

| **NORMOTENSIVE RAT non-invasive** |  |  |  |  |  |
| --- | --- | --- | --- | --- | --- |
| **Author** | **Strain (n)** | **SBP** | **DBP** | **MAP** | **Method** |
| Khazaeli et al., 2023 [15] | SD (6) | 115.1 ± 10.6 |  |  | tail cuff |
| Fu et al., 2023 [16] | Dahl salt-sensitive (38) | 131.8 ± 0.7 | 90.2 ± 1.0 |  | tail cuff |
| Candido et al., 2023 [17] | WKY (18) |  |  | 109 ± 2 | tail cuff |
| Shen et al., 2023 [18] | SD (10) |  |  | 85.90 ± 2.60 | tail cuff |
| Ahad et al., 2023 [19] | WKY (5) | 110.60 ± 3.32 |  |  | tail cuff |
| Kolesnyk et al., 2023 [20] | WKY (30) | 115 ± 1.8 | 68.1 ± 1.2 | 83.8 ± 0.9 | tail cuff |
| Monteiro et al., 2023 [21] | WKY (6) | 122.5 ± 2.2 |  |  | tail cuff |
| Wang et al., 2023 [22] | WKY (3) | 113.35  ± 6.89 |  |  | tail cuff |
| Mohammed et al., 2023 [23] | WKY (8) | 123.55 | 85.51 |  | tail cuff |
| Matsumoto et al., 2023 [24] | WKY (8) | 119 ± 3 |  |  | tail cuff |
| Moke et al., 2023 [25] | WKY ( 5) | 89.67 ± 0.33 | 66.00 ± 0.00 | 74.00 ± 0.00 | tail cuff |
| Ma et al., 2023 [26] | WKY (15) | 118 ± 6.87 | 95.64 ± 2.15 |  | tail cuff |
| Teng et al., 2023 [27] | SD (6) | 120.51 ± 5.28 | 70.12 ± 4.58 |  | tail cuff |
| Maneesai et al., 2023 [28] | SD (8) | 115.08 ± 2.84 | 73.28 ± 4.41 | 87.21 ± 3.81 | tail cuff |
| Goto et al., 2023 [29] | SD (10) | 118 ± 2 |  |  | tail cuff |
| Ahmed et al.. 2023 [30] | WKY (6) | 123.0 ±1.5 | 67.6 ± 1.0 | 84.1 ± 1.6 | tail cuff |
| Chen et al., 2023 [31] | WKY (15) | 119.93 ± 7.16 | 96.96 ± 6.44 |  | tail cuff |
| Yuan et al., 2023 [32] | WKY (8) | 81.72 ± 23.50 | 67.10 ± 10.87 | 72.65 ± 16.15 | tail cuff |
| Gonzales et al., 2023 [33] | WKY (5) | 104 ± 4 |  |  | tail cuff |
| Kalkan et al., 2023 [34] | SD (24) | 125 ± 5.1 |  |  | tail cuff |
| Shamardl et al., 2023 [35] | WKY (24) | 114.88 ± 3.94 | 82.75 ± 2.60 |  | tail cuff |
| Baka et al., 2023 [36] | WKY (28) | 126.10 ± 0.73 |  |  | tail cuff |
| Alanazi et al., 2023 [37] | WKY (48) | 126,45 ± 1,46 | 86,37 ± 1,46 | 99,41 ± 1,41 | tail cuff |
| Gomes et al., 2023 [38] | Zucker lean (8) | 131.3 (± 2.9) | 73.44 (± 8.3) |  | tail cuff |
| Gonçalves et al., 2023 [39] | WKY (8) | 138 ± 2.8 |  |  | tail cuff |
| Desplanche et al., 2023 [40] | WKY (20) |  |  | 102.3 ± 1.5 | tail cuff |
| Lim et al., 2024 [41] | WKY (8) | 138.0 ± 16.9 |  |  | tail cuff |
| Forester et al., 2024 [42] | SD (6) | 138 ± 5 |  |  | tail cuff |
| McCalla et al., 2024 [43] | SD (10) | 120 ± 1 | 81 ± 1 | 94 ± 1 | tail cuff |
| Zhang et al., 2024 [44] | SD (60) |  |  | 115.82 ± 5.17 | tail cuff |
|  |  |  |  |  |  |
| **HYPERTENSIVE RAT non-invasive** |  |  |  |  |  |
| **Author** | **Strain (n)** | **SBP** | **DBP** | **MAP** | **Method** |
| Candido et al., 2023 [17] | SHR (18) |  |  | 170 ± 4 | tail cuff |
| Wang et al., 2023 [22] | SHR (3) | 143.53 ± 18.88 |  |  | tail cuff |
| Mohammed et al., 2023 [23] | SHR (32) | 191.19 | 142.63 |  | tail cuff |
| Chen et al., 2023 [31] | SHR (15) | 186.24 ± 6.56 | 139.5 ± 4.91 |  | tail cuff |
| Yuan et al., 2023 [32] | SHR (8) | 176.21 ± 17.03 | 138.67 ± 33.06 | 121.72 ± 15.03 | tail cuff |
| Gonçalves et al., 2023 [39] | SHR (8) | 186 ± 5.1 |  |  | tail cuff |
| Lim et al., 2024 [41] | SHR (8) | 179.8±16.9 |  |  | tail cuff |
| Jo et al., 2023 [45] | SHR (4) | 206.6 ± 2.8 | 159.2 ± 7.3 |  | tail cuff |
| Liu et al., 2023 [46] | SHR (14) | 199.43 ± 28.33 | 156.14 ± 29.75 |  | tail cuff |
| Yan et al., 2023 [47] | SHR (8) | 189 ± 3 | 158 ± 4 | 170 ± 3 | tail cuff |
| Corrêa et al., 2023 [48] | SHR (10) | 205.2 ± 6.1 |  |  | tail cuff |
| Castoldi et al., 2023 [49] | SD (7) | 142.9 ± 3.0 |  |  | tail cuff |
| Liskova et al., 2023 [50] | SHR (8) | 173 ± 3 |  |  | tail cuff |
| D’Ambrosio et al., 2023 [51] | SD (8) | 156.2 ± 5.2 | 94.9 ± 6.4 | 115.3 ± 5.6 | tail cuff |
| Tandirerung et al., 2023 [52] | WKY (15) | 147.78 | 115.17 | 125.78 | tail cuff |
|  |  |  |  |  |  |
| **NORMOTENSIVE RAT invasive** |  |  |  |  |  |
| **Author** | **Strain (n)** | **SBP** | **DBP** | **MAP** | **Method** |
| Raji et al., 2023 [53] | SD (4) | 116.50 ± 3.69 | 63.50 ± 5.06 |  | carotid artery |
| Wu et al., 2023 [54] | SD (24) | 147.4 ± 4.7 | 114.8 ± 7.9 |  | carotid artery |
| Olatoye et al., 2023 [55] | WKY (36) | 123.70 ± 11.44 | 106.25 ± 2.83 | 113.00 ± 3.58 | carotid artery |
| Zhang et al., 2023 [56] | SD (6) | 123.6 ± 1.6 | 79.3 ± 0.4 | 98.6 ± 3.7 | telemetry |
| Gomes et al., 2023 [57] | WKY (4) | 127 ± 1 | 95 ± 2 | 109 ± 1 | telemetry |
| Toczek et al., 2023 [58] | WKY (4) | 125 ± 1  128 ± 5 | 85 ± 1  88 ± 1 | 105 ± 1  107 ± 3 | telemetry |
| Abdulsalam et al., 2023 [59] | WKY (10) |  |  | 85.15 ± 10.6 | abdominal aorta |
| Mohamed et al., 2024 [60] | WKY (10) |  |  | 79 ± 6.9 | abdominal aorta |
|  |  |  |  |  |  |
| **HYPERTENSIVE RAT invasive** |  |  |  |  |  |
| **Author** | **Strain (n)** | **SBP** | **DBP** | **MAP** | **Method** |
| Toczek et al., 2023 [58] | SHR (4) | 176 ± 5  178 ± 2 | 121 ± 4  120 ± 0 | 150 ± 4  150 ± 1 | telemetry |
| Geraldes et al., 2023 [61] | SHR (30) | 161 ± 1 | 133 ± 1 | 142 ± 1 | telemetry |
| Fan et al., 2024 [62] | SHR (6) | 173.7 ± 9.0 |  |  | telemetry |
| Sun et al., 2024 [63] | Dahl salt sensitive (7) | 162.33 ± 3.41 |  |  | telemetry (abdominal aorta) |
|  |  |  |  |  |  |
