# Supplementary material for: Measurement of blood pressure in rats: Invasive or noninvasive methods?
Source: Physiol Rep. 2024 Sep 12;12(17):e70041. doi: 10.14814/phy2.70041 (PMC11392657; doi:10.14814/phy2.70041)
Supplement: Supplementary file 3 — Data S3. [file PHY2-12-e70041-s003.doc]

**Supplement 3 -** Baseline values of BP data (SBP, DBP, MAP) and HR from normotensive and hypertensive sexually mature male rats recorded from awake animals by non-invasive and invasive methods from 2021 to 2023. BP, blood pressure; SBP, systolic blood pressure; DBP, diastolic blood pressure; MAP, mean arterial pressure; HR, heart rate; n, number of evaluated rats for mean BP data and HR; BN, Brown-Norway rat; DOCA-salt, deoxycorticosterone acetate-salt rat; SD, Sprague-Dawley rat; WKY, Wistar or Wistar-Kyoto strain; SHR, Spontaneously Hypertensive rat.

| **NORMOTENSIVE RAT  non-invasive** |  |  |  |  |  |  |
| --- | --- | --- | --- | --- | --- | --- |
| **Author** | **Strain (n)** | **SBP** | **DBP** | **MAP** | **HR** | **Method** |
| Candido et al., 2023 [17] | WKY (18) |  |  | 109 ± 2 | 352 ± 5 | tail-cuff |
| Wang et al., 2023 [22] | WKY (3) | 113.35  ± 6.89 |  |  | 398.53 ± 30.02 | tail-cuff |
| Moke et al., 2023 [25] | WKY (5) | 89.67 ± 0.33 | 66.00 ± 0.00 | 74.00 ± 0.00 | 355.33 ± 18.48 | tail-cuff |
| Maneesai et al., 2023 [28] | SD (8) | 115.08 ± 2.84 | 73.28 ± 4.41 | 87.21 ± 3.81 | 344.34 ± 10.42 | tail-cuff |
| Ahmed et al., 2023 [30] | WKY (6) | 123.0 ± 1.5 | 67.6 ± 1.0 | 84.1 ± 1.6 | 299.4 ± 11.6 | tail-cuff |
| Gonzales et al., 2023 [33] | WKY (5) | 104 ± 4 |  |  | 280 ± 7 | tail-cuff |
| Shamardl et al., 2023 [35] | WKY (24) | 114.88 ± 3.94 | 82.75 ± 2.60 |  | 421.33 ± 3.98 | tail-cuff |
| Baka et al., 2023 [36] | WKY (28) | 126.10 ± 0.73 |  |  | 384.27 ± 2.84 | tail-cuff |
| Desplanche et al., 2023 [40] | WKY (20) |  |  | 102.3 ± 1.5 | 357 ± 4 | tail-cuff |
| Del Mauro et al., 2021 [64] | WKY (8) | 131 ± 2 |  |  | 324 ± 7 | tail-cuff |
| Hashemi et al., 2021 [65] | WKY (6) | 112.65 ± 6.21 |  |  | 328 ± 8.27 | tail-cuff |
| Fauss et al., 2021 [66] | SD (8) | 106.15 |  |  | 257.5 | tail-cuff |
| Bian et al., 2021 [67] | WKY (18) | 123.43 ± 2.88 | 80.59 ± 2.01 | 94.87 ± 1.71 | 365.54 ± 6.34 | tail-cuff |
| Luo et al., 2021 [68] | WKY (12) | 106.5 | 73.9 | 83.2 | 377.3 | tail-cuff |
| Bin Jardan et al., 2021 [69] | WKY (6) | 118 ± 8 | 74 ± 12 | 88 ± 7 | 357 | tail-cuff |
| Huang et al., 2022 [70] | Zucker lean (n = 8) | 120 ± 3.5 |  |  | 366.8 ± 4.9 | tail-cuff |
| Dantas et al., 2021 [71] | WKY (6) | 151 | 115 |  | 391 | tail-cuff |
| Lezama-Martinez et al., 2021 [72] | WKY (6) |  |  | 92.5 ± 2.5 | 345.4 ± 14 | tail-cuff |
| Liu et al., 2021 [73] | WKY (6) | 118.7 ± 6.4 |  |  | 350 ± 13.1 | tail-cuff |
| Alam et al., 2021 [74] | Not specified | 119.0 ± 1.0 | 75.8 ± 0.79 | 90.3 ± 0.62 | 352.5 ± 1.8 | tail-cuff |
| Coatl-Cuaya et al., 2022 [75] | WKY (19) | 152.2 ± 2.9 | 103.8 ± 2.5 |  | 283.8 ± 7.8 | tail-cuff |
| Maneesai et al., 2022 [76] | SD (8) | 114.67 ± 2.22 | 74.52 ± 2.42 | 90.21 ± 5.07 | 344.00 ± 5.03 | tail-cuff |
| Ito et al., 2021 [77] | SD (10) | 117 ± 4 |  |  | 381 ± 16 | tail-cuff |
| Ojetola et al., 2021 [78] | WKY (5) | 130.2 ± 3.2 | 99.0 ± 7.8 |  | 272.8 ± 23.7 | tail-cuff |
| Ojetola et al., 2021 [79] | WKY (5) | 136.40 ± 2.16 | 97.40 ± 6.46 | 110.40 ± 3.9 | 271.00 ±19.99 | tail-cuff |
| Batool et al., 2022 [80] | SD (5) | 123.17 ± 2.14 | 103.63 ±2.99 | 113.35±1.55 | 363.13±15.67 | tail-cuff |
| Amer et al., 2022 [81] | WKY (8) | 84.33 ± 4.68 | 64.17 ± 4.01 |  | 401.00 ± 7.09 | tail-cuff |
| Sunagawa et al., 2021 [82] | Dahl Salt-resistant  (5)  Dahl-Salt senzitive  (8) | 104 ± 1  111 ± 3 | 62 ± 9  67 ± 9 |  | 439 ± 8  416 ± 22 | tail-cuff |
| Jan-On et al., 2021 [83] | SD  (8) | 123.1 ± 5.4 | 80.5 ± 4.5 | 89.3 ± 12.7 | 360 ± 21 | tail-cuff |
| Hashmi et al., 2021 [84] | WKY  (6) | 117 ± 5 |  | 99 ± 4 | 326 ± 15 | tail-cuff |
| Torok et al., 2021 [85] | WKY (6) | 114.8 ± 2.6 |  |  | 412.3 ± 5.0 | tail-cuff |
| Aali et al., 2021 [86] | WKY (7) | 119.62±2.90 |  |  | 227.21±19.6 | tail-cuff |
| Pan et al., 2021 [87] | WKY (6) | 118.4 ± 4 | 80.3 ± 2.8 |  | 373.7 ± 13.3 | tail-cuff |
| Chia et al., 2021 [88] | WKY (8) | 115±1 |  |  | 300±11 | tail-cuff |
| Badr et al., 2021 [89] | Albino (4) | 120 ± 4 | 103 ± 3 | 108 ± 4 | 352 ± 23 | tail-cuff |
| Hsieh et al., 2021 [90] | WKY (8) | 112 ± 4 | 89 ± 5 | 95 ± 4 | 277 ± 10 | tail-cuff |
| **Afzal et al., 2021 [91]** | WKY (6) | 118 ±  5 | 79 ± 3 | 92 ± 8 | 312 ± 10 | tail-cuff |
| Baskaran et al., 2022 [92] | WKY (8) | 147.3 ± 12.09 | 106.0 ± 5.29 | 116.6 ± 5.13 | 328.8 ± 14.70 | tail-cuff |
| Grigorova et al., 2022 [93] | SD (18) | 117 ± 2 |  |  | 388 ± 10 | tail-cuff |
| Li et al., 2022 [94] | SD (8) | 125.8±4.37 | 91.7±1.42 |  | 365.7±15.4 | tail-cuff |
| Iampanichakul et al., 2022 [95] | SD (8) | 122 ± 3.97 | 79.89 ± 4.42 | 93.93 ± 4.20 | 361.89 ±16.53 | tail-cuff |
| Nguyen et al., 2022 [96] | WKY (12) | 121.26 ±16.67 | 100.97 ± 3.99 | 109.33 ±15.1 | 431.45 ±26.36 | tail-cuff |
| Pauziene et al., 2022 [97] | WKY (8) | 133 ± 6 | 109 ± 5 | 117 ± 5 | 175 ± 9 | tail-cuff |
| Anamalley et al., 2022 [98] | WKY (8) | 124.40 ±1.93 | 85.93 ±2.06 | 98.75 ±1.92 | 366.64 | tail-cuff |
| Zou et al., 2022 [99] | SD (8 per group) | 94.57 ± 6.24  99.00 ± 8.00 | 61.65 ± 7.03  58.20 ±12.45 |  | 361.30 ±28.29  353.72 ± 8.87 | tail-cuff |
| Draginic et al., 2022 [100] | Dark Agouti (10) | 133.6 ± 7.6 | 81.4 ± 4.93 |  | 367.60 ± 17.62 | tail-cuff |
| Ahmad 2022 [101] | SD (6) | 119 ± 7 | 89 ± 5 | 99 ± 4 | 325 ± 10 | tail-cuff |
| Simko et al., 2022 [102] | WKY (15) | 131.71 ± 3.71 |  |  | 375.93 ± 11.61 | tail-cuff |
| Mendes et al., 2022 [103] | WKY (10) | 115.4 ± 4.2 |  |  | 400.1 ± 30.7 | tail-cuff |
| Prasad et al., 2022 [104] | WKY (6) | 116 ± 8.94 | 72.13 ± 5.42 | 86.4 ± 6.33 | 347 ± 26.14 | tail-cuff |
| García-Pedraza et al., 2022 [105] | WKY (12) | 124 ± 4 |  |  | 330 ± 15 | tail-cuff |
| Hong et al., 2022 [106] | SD (6) | 154.7 ± 3.2 | 69.7 ± 1.5 |  | 422.0 ± 13.1 | tail-cuff |
| **Nguelefack-Mbuyo et al., 2022 [107]** | WKY (9) | 118.60 ± 0.34 | 78.55 ± 0.49 |  | 358.60 ± 9.11 | tail-cuff |
| Lei et al., 2023 [108] | WKY (27) |  |  | 120 ± 11 | 345 ± 25 | unspecified |
|  |  |  |  |  |  |  |
| **HYPERTENSIVE RAT  non-invasive** |  |  |  |  |  |  |
| **Author** | **Strain (n)** | **SBP** | **DBP** | **MAP** | **HR** | **Method** |
| Candido et al., 2023 [17] | SHR (18) |  |  | 170 ± 4 | 367 ± 7 | tail-cuff |
| Wang et al., 2023 [22] | SHR (3) | 143.53 ± 18.88 |  |  | 447.67 ± 26.76 | tail-cuff |
| Liskova et al., 2023 [50] | SHR (8) | 173 ± 3 |  |  | 500 ± 11 | tail-cuff |
| Bian et al., 2021 [67] | SHR (18) | 185.93 ± 3.39 | 150.03 ±4.34 | 162.0 ± 3.23 | 358.45 ± 5.53 | tail-cuff |
| Luo et al., 2021 [68] | SHR (12) | 163.8 | 121.9 | 130 | 461.8 | tail-cuff |
| Bin Jardan et al., 2021 [69] | WKY (6) | 176 ± 17 | 102 ± 25 | 126 ± 19 | 320 | tail-cuff |
| Lezama-Martinez et al., 2021 [72] | SHR (6) |  |  | 141.8 ± 5.5 | 406 ± 4.8 | tail-cuff |
| Liu et al., 2021 [73] | SHR (6) | 187.6 ± 5.7 |  |  | 353.4 ± 12.3 | tail-cuff |
| Coatl-Cuaya et al., 2022 [75] | SHR (20) | 187.6 ± 3.2 | 140.1 ± 3.2 |  | 337.4 ± 9.3 | tail-cuff |
| Pan et al., 2021 [87] | SHR (6) | 204.4 ± 3.9 | 162 ± 4.6 |  | 439.9 ± 8.2 | tail-cuff |
| Hsieh et al., 2021 [90] | SHR (8) | 194 ± 2 | 156 ± 2 | 169 ± 2 | 387 ± 16 | tail-cuff |
| **Afzal et al., 2021 [91]** | SHR (6) | 159 ±  4 | 119 ±  6 | 132 ± 5 | 386 ± 9 | tail-cuff |
| Baskaran et al., 2022 [92] | SHR (8) | 212.0 ± 7.48 | 175.3 ± 8.95 | 188.6 ± 9.46 | 423.0 ± 5.16 | tail-cuff |
| Pauziene et al., 2022 [97] | SHR (8) | 195 ± 7 | 149 ± 5 | 164 ± 7 | 254 ± 26 | tail-cuff |
| Simko et al., 2022 [102] | SHR (15) | 182.89 ± 4.22 |  |  | 474.95 ± 10.53 | tail-cuff |
| Ajamu et al., 2021 [109] | Dahl salt-sensitive (12) | 162 ± 13 |  |  | 418 ± 23 | tail-cuff |
| Lee et al., 2021 [110] | Dahl salt-sensitive (4) | 172 ± 8 | 124 ± 8 |  | 372 ± 16 | tail-cuff |
| Matsuoka et al., 2021 [111] | SHR (10) | 204.0 ± 5.8 | 159.3 ± 3.9 | 173.3 ± 4.3 | 412.3 ± 18.5 | tail-cuff |
| El Maleky  et al., 2021 [112] | WKY (6) | 151.2 ± 1.7 | 137 ± 2.4 | 141.7 | 345 ± 2.9 | tail-cuff |
| Soltani Hekmat et al., 2021 [113] | SD (unspecified) | 199.3±19.3 | 130.7± 14.9 |  | 407.4±55.5 | tail-cuff |
| Rassler et al., 2022 [114] | SHR (12) | 173.8 ± 3.1 |  |  | 384.2 ± 10.3 | tail-cuff |
| Nakatsukasa et al., 2022 [115] | Spontaneously Hypertensive Heart Failure Rat (7) | 152.0±3.2 | 103.3±3.1 |  | 336.8±10.2 | tail-cuff |
| Kluknavsky et al., 2022 [116] | SHR (11) | 146 ± 3 |  |  | 531 ± 18 | tail-cuff |
|  |  |  |  |  |  |  |
| **NORMOTENSIVE RAT – invasive** |  |  |  |  |  |  |
| **Author** | **Strain (n)** | **SBP** | **DBP** | **MAP** | **HR** | **Method** |
| Gomes et al., 2023 [38] | WKY (4) | 127 ± 1 | 95 ± 2 | 109 ± 1 | 378 ± 31 | telemetry  (femoral artery) |
| Olatoye et al., 2023 [55] | WKY (36) | 123.70 ± 1.44 | 106.25 ± 2.83 | 113.00 ± 3.58 | 390.70 ± 13.13 | carotid artery |
| Zhang et al., 2023 [56] | SD (6) | 123.6 ± 1.6 | 79.3 ± 0.4 | 98.6 ± 3.7 | 378 ± 7 | telemetry |
| Griffiths et al., 2021 [117] | WKY (17 per group) | 127 ± 3  127 ± 5 | 83 ± 3  87 ± 5 | 103 ± 3  104 ± 5 | 312 ± 5  314 ± 7 | radiotelemetry |
| Selejan et al., 2022 [118] | SD (5) |  |  | 118 ± 10 | 350 ± 11 | telemetry |
| Barrera et al., 2021 [119] | SD (8 and 11) | 118 ± 6  114 ± 4 | 103 ± 7  98 ± 6 |  | 383 ± 42  399 ± 24 | telemetry  (abdominal aorta) |
| Potter et al., 2021 [120] | BN (16) |  |  | 94±1 | 374±7 | telemetry  (abdominal aorta) |
| [Ayaz](https://www.frontiersin.org/people/u/1292749) et al., 2021 [121] | SD (4) | 118.15 ± 0.76 |  | 100.25 ± 0.59 | 401.86 ± 2.62 | telemetry  (abdominal aorta) |
| Das et al., 2022 [122] | SD (8) |  |  | 107.4 ± 1.9 | 401 ± 10 | telemetry  (distal aorta) |
| Costa-Ferreira et al., 2021 [123] | WKY (8) |  |  | 111 ± 3 | 408 ± 18 | abdominal aorta |
| Oliveira et al., 2021 [124] | WKY (7 and 9) |  |  | 105 ± 6  107 ± 4 | 365 ± 8  369 ± 8 | abdominal aorta |
| Silva et al., 2021 [125] | WKY (8) |  |  | 118 ± 8 | 342 ± 9 | abdominal aorta |
| Flahault et al., 2021 [126] | SD (≥ 4) |  |  | 112 ± 1 | 405 ± 8 | femoral artery |
| Cruz et al., 2021 [127] | WKY (8) |  |  | 110 ± 2 | 338 ± 6 | femoral artery |
| dos Santos et al., 2021 [128] | WKY (10) |  |  | 94 ± 3 | 355 ± 9 | femoral artery |
| Sharma et al., 2021 [129] | SD (6) |  |  | 78±4 | 271±12 | femoral artery |
| Barretto-de-Souza et al., 2021 [130] | WKY (8) |  |  | 108 ± 2.3 | 342 ± 9 | femoral artery |
| Lopes et al., 2022 [131] | WKY (5) |  |  | 103 ± 6 | 412 ± 35 | femoral artery |
| [Sedighi](javascript:;) et al., 2021 [132] | WKY (8) | 100.71 ± 5.75 | 80 ± 6.81 |  | 141 ± 13.77 | femoral artery |
| Oliveira et al., 2022 [133] | WKY (6 per group) |  |  | 102 ± 3  102 ± 2 | 357 ± 9  354 ± 8 | femoral artery |
| Fioretti et al., 2022 [134] | WKY (6) |  |  | 118 ± 3 | 334 ± 13 | femoral artery |
| Luz et al., 2022 [135] | WKY (7) | 138 ± 7 | 104 ± 5 | 119 ± 2 | 374 ± 11 | femoral artery |
| dos Santos et al., 2022 [136] | WKY (4) |  |  | 105.1 ± 2.8 | 350.6 ± 11.6 | femoral artery |
| Kirillov et al., 2021 [137] | WKY (40) | 113.9±10.75 | 96.1±10.35 | 108.6±25.27 | 431.4±34.72 | femoral artery |
| Zicha et al., 2021 [138] | Dahl-Salt senzitive (10) |  |  | 115 ± 3 | 367 ± 19 | carotid artery |
| Komnenov and Rossi 2023 [139] | SD (12) |  |  | 108 ± 3 | 341 ± 12 | carotid artery |
|  |  |  |  |  |  |  |
| **HYPERTENSIVE RAT invasive** |  |  |  |  |  |  |
| **Author** | **Strain (n)** | **SBP** | **DBP** | **MAP** | **HR** | **Method** |
| Griffiths et al., 2021 [117] | SHR (17 per group) | 169 ± 4  181 ± 3 | 106 ± 4  117 ± 3 | 138 ± 3  147 ± 2 | 306 ± 3  308 ± 2 | radiotelemetry |
| Selejan et al., 2022 [118] | SHR (5) |  |  | 190 ± 6 | 325 ± 12 | telemetry |
| Gardim et al., 2021 [140] | SHR (9) | 191 ± 1 | 152 ± 3 | 165 ± 2 | 379±5 | femoral artery |
| Marc et al., 2021 [141] | DOCA-salt (6) |  |  | 193 ± 9 | 386 ± 21 | femoral artery |
| Bandoni et al., 2021 [142] | SHR (5) | 204 ± 8.2 | 144 ± 8.4 | 172 ± 8.1 | 376±28.1 | femoral artery |
| Oleksa et al., 2021 [143] | SHR (6-7) |  |  | 184 ± 3 | 361 ± 10 | carotid artery |
|  |  |  |  |  |  |  |
